# Acknowledgement to Reviewers of *Dentistry* *Journal* in 2017

**DOI:** 10.3390/dj6010003

**Published:** 2018-01-19

**Authors:** Dentistry Journal Editorial Office

**Affiliations:** MDPI AG, St. Alban-Anlage 66, 4052 Basel, Switzerland; dentistry@mdpi.com

Peer review is an essential part in the publication process, ensuring that *Dentistry Journal* maintains high quality standards for its published papers. In 2017, a total of 36 papers were published in the journal. Thanks to the cooperation of our reviewers, the median time to first decision was 25 days and the median time to publication was 74 days. The editors would like to express their sincere gratitude to the following reviewers for their time and dedication in 2017:

Abduo, JaafarLauritano, DorinaAgnello, MelissaLlena, CarmenAgrawal, PriyankaLoewy, ZviAmarasena, NajithLopez Jornet, PiaBaba, Nadim Z.Lops, DiegoBagby, MichaelMaiorana, CarloBarberis, FabrizioMauldon, EmilyBartlett, John D.Meldrum, AlisonBelibasakis, Georgios N.Meurman, JukkaBennani, VincentMilosevic, AlexBlum, Igor RNieminen, PenttiBorghaei, Ruth C.Okumura, NobuakiBotelho, Michael GeorgeOshima, HayatoCannell, PhillipPalomo, LeenaCarpenter, GuyPapadopoulou, AlexandraChass, Gregory AdamPark, Young-BumChen, JunningPark, Jae HyunChieruzzi, ManilaPerry, Jamie L.D’Amario, MaurizioPirani, ChiaraDarvell, BrianPommer, BernhardDelgado Ruiz, RafaelQuock, RyanDo, ThuyRanjitkar, SarbinEramo, StefanoRimondini, LiaFabbrocini, GabriellaRobledo-Sierra, JairoFan, Pui L.Roggendorf, Matthias JFarronato, DavideRoh, SanghoFerreira, Manuel MarquesRuhl, StefanFigueiral, Maria HelenaSagheb, KeyvanFine, James BurkeSaha, ShyamaliFranzén, C.Sarmast, Nima D.Garner, Angelia D.Sato, TakuichiGe, XuepingSchmidt, JochenGherlone, EnricoScribante, AndreaGregory, Richard LSefat, FarshidHach, MariaShahid, SaroashHalazonetis, Demetrios J.Shieh, Tzong-MingHarding, MaireadSinghrao, Sim K.Hardt, MarkusSkapetis, TonyHennyey, DonnaSong, Linyong (Leon)Hmaidouch, RimSoueidan, AssemHoefert, SebastianSperber, Geoffrey H.Hortsch, MichaelSukumaran, AnilHughes, Christopher V.Takamizawa, ToshikiIrie, MasaoTallarico, MarcoJeng, Jiiang-HueiTarro, GiulioJu, XiangqunTroedhan, AngeloKalenderian, ElsbethTulunoglu, IbrahimKamitani, ShigekiWang, ChangxiangKapellas, KostasWertz, Philip W.Kellesarian, SergioWu, Shih-ChingKim, Young-jaeXu, XiaomingKim, Seong-GonYang, Jen-ChangKoizumi, HiroyasuYu, QingsongKosuke, NozakiYuan, KuoKumar, SanthoshYumoto, HiromichiKwon, Tae-yubZhan, LingLam, Otto Lok Tao


